# The effect of *“maviz*ˮ on memory improvement in university students: A randomized open-label clinical trial

**Published:** 2020

**Authors:** Reza Mirheidary, Seyyed Saeed Esmaeili Saber, Mohammad Reza Shaeiri, Mohammad gholami fesharaki

**Affiliations:** 1 *Department of Iranian Traditional Medicine, Faculty of Medicine, Shahed University, Tehran, Iran*; 2 *Department of Clinical Psychology, Faculty of Humanities, Shahed University, Tehran, Iran*; 3 *Department of Biostatistics, Faculty of Medical Sciences, Tarbiat Modares University* *,* *Tehran, Iran*

**Keywords:** Memory, Grapes, “Mavizˮ, Vitis vinifera, Iranian Traditional Medicine

## Abstract

**Objective:**

Numerous studies demonstrated the effect of grape on memory improvement. According to Iranian traditional medicine, “mavizˮ as a specific type of dried grapes can effectively improve memory. However, there is no reported clinical trial on the effect of “mavizˮ on memory improvement in humans. Hence, this study was conducted to investigate “mavizˮ effect on memory in university students.

**Materials and Methods:**

This randomized open-label clinical trial was conducted on a total number of 53 students of Shahed University, Tehran, Iran, from November 2017 to February 2018. The participants were randomly allocated into an intervention group (receiving “mavizˮ, 25 g in the morning for 4 weeks) or a control group (who did not take “mavizˮ). The Digit Span Task and the N-Back Task were used for the measurement of working memory at the pre- and post-intervention stages.

**Results:**

According to the results, no significant differences were found between the two groups regarding age, gender, marital status, and initial working memory test scores. “mavizˮ consumption produced a highly significant improvement in total working memory score in the Digit Span Task (5.18 vs. 2.35, p<0.001) and Acoustic Memory Span (1.29 vs. 0.62, p=0.021). Moreover, “mavizˮ consumption significantly increased the percentage of true responses in the N-Back Task and reduced the mean reaction time in the first level of the task.

**Conclusion:**

“mavizˮ consumption was improved working memory in young healthy adults.

## Introduction

Cognition refers to a group of brain processes that can be defined by means of concepts such as intelligence, memory, and mood (Pribis and Shukitt-Hale, 2014 ▶). The processes of cognition and correct brain activity are unquestionably related to proper nutrition. In this respect, a group of foods or part of them with disease prevention and treatment effects are known as nutraceuticals. Moreover, evidence has been provided that some fruits such as grapes (*Vitis vinifera *L*.*) are endowed with beneficial nutraceutical values for the brain and cognitive processes. Therefore, regular consumption of grapes and its related products can improve memory and play a very important role against neurodegenerative diseases such as Alzheimer’s disease (AD), cancer, cardiovascular diseases, platelet aggregation, atherosclerosis, blood pressure, viral infections, wound and pain (Keservani et al., 2016 ▶; Emami et al., 2010 ▶; Krikorian et al. 2010 ▶; Nayak et al., 2010 ▶).

The beneficial effects of grapes and its relevant products such as grape juice, grape seed extract, and dried grape might be associated with their bioactive phytochemicals including polyphenolic compounds (flavonoids, anthocyanins, resveratrol), tannins (proanthocyanidins), alkaloid compounds (indolamine, melatonin, and serotonin), furfural (a carbohydrate), boron, iron, potassium, calcium, and vitamins ( Iriti and Faoro, 2009 ▶; Iriti and Faoro, 2006 ▶; Iriti et al., 2006 ▶). 

Nowadays, the importance of such compounds for body health and disease prevention is highlighted. Moreover, neurodegenerative diseases, chronic cardiovascular diseases, cancer, and inflammatory diseases can be considered direct results of oxidative stress. During the development of diseases, reactive oxygen species (ROS) are also created and they can trigger a chain of destructive reactions that damage cell membranes such as the membrane of neurons. To neutralize ROS, the body needs antioxidants. Polyphenolic and non-polyphenolic compounds available in grape, can correspondingly play an antioxidative role and protect against these substances (Fodor et al., 2018 ▶; Sheng et al, 2018 ▶; Shukitt-Hale et al., 2005 ▶). 

Resveratrol in grape, can effectively fight against free radicals and inflammatory chains. Animal and human studies showed its beneficial effects on neurodegenerative diseases. In a study, resveratrol was suggested to reduce the extracellular accumulation of beta-amyloid peptides via activating autophagy (Fodor et al., 2018 ▶; Uddin et al., 2018 ▶).

Anthocyanin (a pigment) in grape can pass through the blood-brain barrier, enter the central nervous system, and accumulate in brain regions including temporal lobe gyrus, cortex, and parts of the hippocampus which are areas that are associated with cognitive functions and memory improvement (Krikorian et al., 2010 ▶).

Alkaloid compounds found in grape can inhibit the enzyme acetylcholineesterase (AChE) and maintain the level of acetylcholine in neuronal synapses in order to improve memory and attenuate symptoms of AD. Acetylcholine similarly plays a major role in improving memory and learning. The antioxidative and protective role of alkaloids against free radicals was previously confirmed (Iriti et al., 2006 ▶; Konrath et al., 2013 ▶; Mak and Dickens, 1991 ▶).

Moreover, furfural is recognized as one of the non-polyphenolic compounds of dried grapes produced through dehydration of certain sugars. These compounds have antioxidative and anti-inflammatory properties. For example, in an animal study, it was revealed that the given compound could improve memory and prevent memory impairment (Bakhtiyari et al., 2017 ▶; Kim et al., 2011 ▶). 

Boron, another compound found in grape, was found to be beneficial for brain functioning, memory, and attention (Ghorbanian et al., 2018 ▶).

Flavonoids as important polyphenolic compounds in grape can similarly protect nerve cells and alleviate learning and memory disorders during aging (Lakshmi, et al., 2014 ▶; Rendeiro et al., 2009 ▶).

Raisin, as a kind of dried grape, is rich in polyphenolic compounds and its antioxidant and protective properties were previously reported. In this respect, an animal study showed that antioxidants available in raisins could improve passive avoidance learning and spatial performance (Gol et al. , 2019 ▶; Ghorbanian et al., 2018 ▶).

It should be noted that two products are obtained by grape drying. One is raisin (Currant or “*keshmesh*ˮ) and the other is “mavizˮ which differs from raisin due to the presence of seed and undergoes a different process of grape drying (Hakim Moemen, 2011 ▶; Abounasri Heravi, 1977 ▶). “mavizˮ consists of three parts: pulp, peel, and seed and the best type of “mavizˮ has more flesh and fewer seeds (Hakim Moemen, 2011 ▶). 

In Iranian traditional medicine (ITM), nutrition is the most important factor among essential health principles (Tajadini, Choopani and Saifadini, 2016 ▶). ITM also appreciates “mavizˮ as a medicinal food (At -tabib Esfarayeny, 2014 ▶). This means that “mavizˮ has both nutritional value and medicinal effects which make it as one of the most valuable foodstuffs.

ITM scholars also mentioned memory improvement and anti-forgetfulness effect of consumption of “mavizˮ in the morning (Nazem Jahan, 2008 ▶; Rabban, 1938 ▶). Moreover, benefits and adverse reactions for every part of “mavizˮ have been similarly highlighted. For memory improvement, “mavizˮ seeds must be removed before consumption to reduce adverse reactions and increase therapeutic effects (Aghili Alavi khorasani, 2009 ▶; Bakhtiyari et al., 2017 ▶; Hakim Moemen, 2011 ▶; Nazem Jahan, 2008 ▶). 

There are numerous medical formulas in ITM books for memory improvement and treatment of dementia in which, “mavizˮ consumption, alone or in combination with other herbal medicines, has been advised (Nazem Jahan, 2008 ▶)

In religious texts, Prophet Mohammad has also reiterated that “Whoever continues consuming “mavizˮ while fasting, they can grasp perception, memory, and intelligence” (mohammady reyshahri, 2011 ▶).

Today, in folk medicine in Iran, “mavizˮ is recognized as a substance having an anti-forgetfulness property (Bakhtiyari et al., 2017 ▶).

In cognitive science, memory is referred to as a group of psycho-neuronal processes used by a person to encode, store, and retrieve various experiences and perceptions (Atkinson and Nolen-Hoeksema, 2014 ▶). Memory disorder and dementia are referred to as a group of cognitive disorders in which cognitive content is involved (Aminoff et al., 2015 ▶). 

It should be noted that AD is the most common cause of dementia that comprises 60-80% of cases with dementia (Krikorian et al., 2010 ▶). AD also affects approximately 15% of individuals aged 65 years old or older and approximately 45% of those at the age of 85 years old or over. None of the currently available treatments has been shown to reverse existing deficits or to arrest disease progression (Aminoff et al., 2015 ▶). Based on several hypotheses that were proposed for the etiology of AD, the following therapeutic strategies have been adopted:

1. Stabilization of neurotransmitter levels of acetylcholine via inhibiting AChE and monoamine oxidase (MAO) enzymes.
2. Protection of neurons through the neuroprotective properties of antioxidants to inhibit oxidative stress phenomenon.
3. Reduction of production or accumulation of beta-amyloid peptides.

In an animal experience, grape improved memory via correction of the process of amyloid protein precursors formation. Compounds found in grape could also play a role in improving AD symptoms using each of the three treatment strategies (Fodor et al., 2018 ▶; Konrath et al., 2013 ▶; Sheng et al., 2018 ▶; Uddin et al., 2018 ▶). Numerous studies have further demonstrated the effect of grape and its related products on memory improvement. According to these studies, grape are rich in polyphenolic and non-polyphenolic compounds which have antioxidant effects and may protect the brain neurons through decreasing oxidative stress. The improvement in brain cognitive function and memory was also considered to be related to the presence of these compounds in the grape family (Bakhtiyari et al., 2017 ▶; Lamport et al., 2016 ▶; Lian et al., 2016 ▶; Farbood et al., 2016 ▶; Krikorian et al., 2012 ▶; Emami et al., 2010 ▶; Krikorian et al., 2010 ▶; Joseph, Shukitt-Hale and Willis, 2009 ▶). 

Only one animal trial evaluated the effect of “mavizˮ on memory improvement in rats. In this study, Bakhtiyari et al. mentioned that non-polyphenolic compounds in “mavizˮ with their marked antioxidant effects were responsible for improving memory (Bakhtiyari et al., 2017 ▶). However, the effect of “mavizˮ on memory improvement in human cases especially healthy young individuals with university education, has not been investigated.

With regard to the positive effect of grape on memory improvement as highlighted in several studies and the richness of dried grape with beneficial compound for the body and the brain health (Bakhtiyari et al., 2017 ▶; Ghorbanian et al., 2018 ▶) as well as considering the emphasis of ITM on “mavizˮ consumption for memory improvement, the purpose of this study was to investigate the effect of “mavizˮ on memory improvement in university students.

## Materials and Methods

**Study design**

This randomized open-label clinical trial was conducted on students of Shahed University, Tehran, Iran, from November 2017 to February 2018. 

The inclusion criteria being 18 to 30 years old, being Bachelor’s degree student and having no medical problems affecting neurological functions such as hearing and visual impairments, internal and psychiatric diseases, or a history of epilepsy and head traumas. The exclusion criteria were: adverse reactions to “mavizˮ and unwillingness to continue the study. It should be noted that the individuals were free to withdraw at any time during the study. The block randomization method was used to assign the participants to the intervention or control group. 

After obtaining permission from the Ethics Committee of Shahed University (IR.Shahed.REC.1396.61) and registration of the study in the Iranian Registry of Clinical Trials (IRCT2017062534753N1), students of Shahed University were recruited using a convenient sampling method. To this end, the students were invited to the study via advertising posters and through speeches delivered in university classes. After explaining the study’s objectives, probable risks, benefits of contribution, and the voluntary nature of participation in the study; next, informed consent was obtained and eligible individuals were recruited. Also, students were examined by a physician about the inclusion and exclusion criteria.

**Intervention**

The students were randomly allocated (block randomization) into the intervention (receiving “mavizˮ, 25 g orally in the morning for 4 weeks) or the control group (without “mavizˮ). 

Moreover, the working memory of the students was assessed by the Wechsler Number Memory Scale and N-Back Task at the beginning of the study and after 4 weeks.

Wechsler Number Memory Scale (Digit Span Brain Task):

Digit Span Brain Task is a subtest of the Wechsler memory scale which is one of the most common methods for working memory measurement. The validity of this scale was evaluated and approved using content and convergent methods in a previous study. Also, the reliability of this scale was verified by internal consistency (between 0.74 and 0.93) and test-retest reliability (between 0.62 and 0.82) for all age groups (Groth-Marnat, 2009 ▶; Orangi, Atefvahid, & Ashayeri, 2002 ▶).

This task is based on visual (iconic memory) and acoustic (echoic memory) tests and measures total working memory score and visual and auditory memory span. 

In the visual section, 7 groups of numbers are consecutively presented; after displaying each group of digits, the person must click the digits forward on the monitor with the same sequence. Then, the digits backward begin. In the acoustic section, the person hears the digits through the computer’s acoustic system via headphones.

The total score of working memory is the sum of the forward and backward section scores which can be maximally 28. To calculate this score, the acoustic section is usually used. The reason for choosing acoustic section is that the visual codes disappear faster than acoustic ones in memory (Atkinson and Nolen-Hoeksema, 2014 ▶) however, both acoustic and visual total working memory scores were measured in the present study. Memory span score was also calculated based on the number of memorized digit groups in the forward section which is usually between 5 and 9 in adults.

**N-Back Task:**

This test is employed to evaluate working memory and it is one of the most widely used culture-independent tests. The reliability and validity of the N-Back Task were verified in a previous study. Reliability of the N-Back Task ranges between 0.54 and 0.84 (Nejati, 2013 ▶; Chen et al. 2008 ▶; Jaeggi et al., 2010 ▶; Kane et al, 2007 ▶). 

The N-Back Task used in this study is based on visual memory. This test requires complex attention and measures visual span backward and the ability of number sequencing. In addition, both storage and manipulation of the remembered information occur. In this task, brain cognitive function is assessed during brain activation and it is also used in functional magnetic resonance imaging (fMRI) studies (Owen et al., 2005 ▶). In this test, the series of digits (120 digits) are visually presented one-by-one to the individual, and the person needs to compare the presented digit with N previous digits where N can be 1, 2 or 3. For example, in the series of digits below, if N=2, the person must press button YES for the numbers that are underlined and button NO for other numbers (2 4 8 1 8 9 6 5 4 5 4 9 3 7 3 6 5).

Both tests were performed in a very quiet environment. Before each test, the procedure was explained verbally to the students. The time spent on both tests was about 30 min per person.

“mavizˮ used in this study was obtained from grapes *(Vitis vinifera *L*.*, *Vitaceae *family)*, *Fakhri cultivar, from the city of Maragheh, North West of Iran, and it was then deposited in the Faculty of Pharmacy, University of Tehran, with herbarium code PMP-1625.

There are two methods of grape drying for preparation of “mavizˮ in Iran. In one method, when the grapes are ripe, grape bunches are picked and sun-dried on the soil for 2-3 weeks in order to be converted into “mavizˮ. In the other method, when the grapes are ripe, the ends of bunches of the grapes are cauterized with a hot iron rod so that they are not broken and are retained on the tree until the bunch is semi-dry. Then, the bunches are picked and hanged in shadow in order to be converted into “mavizˮ (Abounasri Heravi, 1977 ▶). The former method is more popular among farmers; however, the latter is preferred in ITM textbooks.

To our knowledge, the present study was the first clinical trial on human cases to investigate memory improvement effect of the normal shape of “mavizˮ. The therapeutic dose of “mavizˮ according to ITM is about 25 g orally per day, but it can be increased up to 90 g. “mavizˮ seeds must be removed manually before consumption by subjects (Bakhtiyari et al., 2017 ▶). Hence, the dosage of 25 g of “mavizˮ per day, was used.

Given that the time for human trials on the effects of antioxidants in healthy humans as well as individuals with cardiovascular diseases was 2-4 weeks (Krikorian et al., 2010 ▶). The intervention period in this study was 4 weeks.

In each visit, the subjects were verbally questioned about their compliance with the intervention. In the first visit, the intervention group received two packages each consisting of seven smaller packs of 25 g of “mavizˮ. Each small pack was to be used every day for two weeks; and in the next visit (two weeks later), two more packages were administered for the remaining two weeks. In the last visit, the individuals of the intervention group were asked for any complications and adverse effects experienced during this period. In the first visit and at the end of four weeks, both intervention and control groups completed working memory tests.

**Outcome**

The primary outcome of this study was improvement of working memory.

**Statistical analysis**

Statistical analyses were performed using SPSS Statistics (version 18) software. In this respect, the Chi-square test was used to compare the categorical variables and t-test and Mann-Whitney U Test were employed to compare continuous ones. Generalized Estimation Equation (GEE) was also utilized to model the correlated and longitudinal data. P-values less than 0.05 were considered statistically significant. Sample size considering Cohen’s d effect size [α=0.05, β=0.1, effect size=0.9, n1=2×n2] was then calculated as 20 and 40 subjects for control and intervention groups, respectively. Considering the possible dropout (10%), it was determined that 48 and 24 subjects should be recruited for intervention and control groups, respectively.

**Figure 1:**
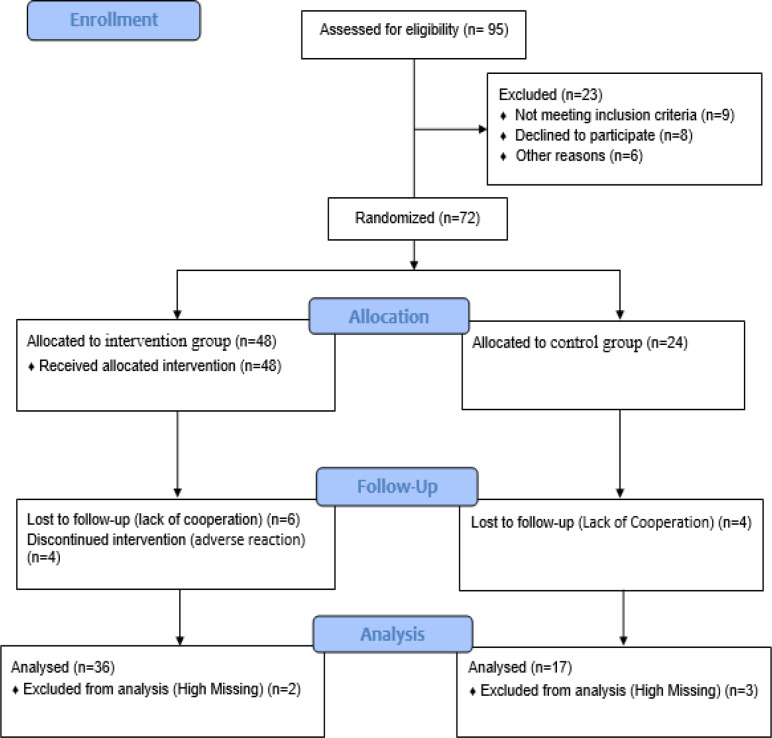
Study flow diagram

In this study, a total number of 95 university students were assessed for eligibility and inclusion criteria; but, 23 were excluded. Thus, 72 university students were recruited and then, randomly allocated by block randomization into intervention (n=48) or control (n=24) groups. Finally, a total number of 53 university students continued until the end of the study (Figure 1).

## Results

This study was conducted on 53 university students (10 (%18.9) males, and 43 (%81.1) females). The distribution of age, gender, and marital status of the study subjects are presented in Table 1. 

According to Table 1, there was no significant difference between the two study groups in terms of age, gender, and marital status. 

Based on students self-declaration, the mean consumption period of “mavizˮ was 22.6 days.

Acoustic and visual total working memory score (A-Score and V-Score), as well as acoustic and visual memory span (A-Span and V-Span) of the subjects of both groups during the study, are shown in Table 2. 

The results indicated an increase in A-Score and V-Score, as well as A-Span and V-Span in the intervention and control groups. However, a highly significant rise in A-Score and A-Span was seen only in the intervention group and the rise in the intervention group, but not in the control group, was significant (p<0.001, p=0.021). However, V-Score and V-Span augmented in the intervention group compared with the control group, the rise was not significant. 

The mean reaction time and the percentage of true responses in the N-Back Task in 3 levels during the study for both groups, are illustrated in Table 3. The results revealed an increase in the percentage of true responses in all 3 levels in both groups. However, only in the first level, the increase in the percentage of true responses in the intervention group was significant compared with that in the control group (p=0.021). Moreover, the results confirmed a decrease in the mean reaction time in all 3 levels in both groups. However, only in the first level, a drop in the mean reaction time in the intervention group was significant compared with that in the control group (p=0.001).

According to Tables 2 and 3, no significant difference was observed between the intervention and control groups in initial test scores. “mavizˮ consumption could also cause adverse reactions such as headache, insomnia, acne, and hematuria in some study subjects. In this respect, headache occurred in 3 individuals after 1-2 weeks of intake and this effect was severe in one person and she discontinued the intervention. Insomnia was seen in one person after one week of consumption and she also withdrew from the intervention. Moreover, acne was reported in 5 subjects during the study; however, in one person with a history of acne, it occurred after three days of intake and he discontinued the intervention. Hematuria was observed in one person after 25 days of consumption but it was resolved upon withdrawal.

**Table1:** Demographic characteristics of the participants

		**Group**				**p-value**
		**Intervention**		**Control**		
		**N**	**%**	**N**	**%**	
**Gender**	male	7	19.4	3	17.6	1.000
	female	29	80.6	14	82.4	
**Marital Status**	Single	31	86.1	16	94.1	0.651
	Married	5	13.9	1	5.9	
**Age (years)**		**Mean**±** SD**		**Mean**±** SD**		
		20.53±1.23		21.35±2.76		0.260

**Table 2:** Acoustic and visual total working memory score (A-Score and V-Score), acoustic and visual memory span (A-Span and V-Span) of the intervention and control groups in the Digit span task

		**Before**	**After**			
	**Group**	**Mean**± **SD**	**Mean**± **SD**	**Diff**	**p-value1**	**p-value2**
**A-Score**	Intervention	13.83±4.26	19.01±3.88	5.18	<0.001	<0.001
	Control	14.18±4.13	16.53±4.07	2.35	0.002	
	P-value	0.723	0.032			
**V-Score**	Intervention	17.89±5.46	21.43±4.76	3.54	<0.001	0.586
	Control	17.41±5.14	20.41±4.39	3.00	0.002	
	P-value	0.667	0.297			
**A- Span**	Intervention	6.08±1.38	7.37±1.04	1.29	<0.001	0.021
	Control	6.24±1.25	6.86±1.66	0.62	0.027	
	P-value	0.750	0.094			
**V- Span**	Intervention	7.22±1.61	7.58±1.32	0.36	0.059	0.376
	Control	6.94±1.64	7.65±1.05	0.71	0.040	
	P-value	0.565	0.823			

P-value: the comparison of values between two groups P-value 1: the comparison of values pre and post-intervention within every group, and P-value 2: the comparison of values between groups pre and post-intervention, SD: Standard deviation

**Table 3:** Mean reaction time and percentage of true response in the N-back task for the intervention and control groups

			**Before**		**After**			
		**Group**	**Mean** ± **SD**		**Mean** ± **SD**		**P- value1**	**P- value2**
	**True. percentage**	Intervention	71.6±0.269		85.4±0.193		0.001	0.021
**Level 1-**		Control	78.9 ± 0.211		90.5±0.128		0.007	
		P-value	0.383			0.291		
	**Reaction time**	Intervention	625.7 ± 153.7		531.8±151.0		<0.001	0.001
		Control	569.0 ± 91.12		554.0±79.9		0.326	
		P-value	0.216			0.616		
	**True. percentage**	Intervention	39.9±0.108		45.5±0.075		<0.001	0.972
**Level 2-**		Control	40.6±0.096		46.1±0.079		0.003	
		P-value	0.827			0.789		
	**Reaction time**	Intervention	786.2±187.80		666.3±184.4		<0.001	0.773
		Control	798.9±180.66		691.6±116.1		0.018	
		P-value	0.831			0.645		
**Level 3-**	**True. percentage**	Intervention	55.9±0.2142		64.4±0.232		0.002	0.841
		Control	61.6±0.1601		67.6±0.192		0.023	
		p-value	0.401			0.648		
	**Reaction time**	Intervention	766.9±183.16		679.9 ± 187.51		<0.001	0.370
		Control	737.5±178.69		676.6±133.05		0.208	
		P-value	0.623			0.952		

P-value: the comparison of values between two groups P-value 1: the comparison of values pre and post-intervention within every group, and P-value 2: the comparison of values between groups pre and post-intervention, SD: Standard deviation

## Discussion

The present trial showed that daily consumption of “mavizˮ for 4 weeks could improve working memory in university students. Considering the results of the Digit Span Task, “mavizˮ could significantly increase the total working memory score and acoustic memory span. Moreover, “mavizˮ could significantly augment the percentage of true responses and decrease mean reaction time in the N-Back Task on the first level.

Reviewing the previous literature showed that no human studies have examined the effect of “mavizˮ on human memory so far. Therefore, the present study was the first one in this regard.

It should be noted that grape and its related products contain polyphenolic and non-polyphenolic compounds with antioxidant properties whose effects on improving memory and preventing memory impairment were demonstrated in multiple clinical trials. Grape-based alkaloids exert beneficial effects in learning and memory processes via inhibiting cholinesterase enzyme and stabilizing acetylcholine in neural synapses. Furfural, mostly found in dried grape particularly “mavizˮ, could also improve memory due to its antioxidant properties (Bakhtiyari et al., 2017 ▶).

In a study done by Ghorbanian et al. in aged rats, oral administration of 6 g of raisin on a daily basis with meals for 90 days, could improve spatial performance and passive avoidance learning, significantly increase the plasma antioxidant power, enhance the number of neurons in the hippocampus and the corpus callosum, and provide more order in the tissue of these areas compared with the control group. This study showed that the neuroprotective property of raisin could improve both working and reference memory functions(Ghorbanian et al., 2018 ▶).

In a study done by Krikorian et al., oral administration of Concord grape juice (CGJ) to older adult subjects with mild cognitive impairment for 16 weeks reduced semantic interference in memory tasks. Moreover, the results of the fMRI along with the N-Back Task showed higher activity in the areas of the anterior and posterior cortex of the right hemisphere in individuals who had received grape juice. It should be noted that Concord grapes are rich in polyphenolic compounds that have proven antioxidant activities (Krikorian et al., 2012 ▶). In another investigation, the use of CGJ by elderly adults with mild cognitive impairment for 12 weeks revealed a significant improvement in verbal learning (Krikorian et al., 2010 ▶). In another study, oral administration of 355 ml CGJ (containing 777 mg of polyphenol) to 40- to 50-year-old working mothers improved immediate spatial memory and performance (Lamport et al., 2016 ▶).

In a trial using animal models of AD, intraperitoneal injection of an aqueous and ethanolic extract of “mavizˮ without seeds for 3 weeks at the doses of 150-300 mg per kg per day, could inhibit memory impairment in rats. Moreover, “mavizˮ could increase the levels of catalase and superoxide dismutase in the hippocampus of the rats (Bakhtiyari et al., 2017 ▶).

All of the above-mentioned investigations showed some kind of memory improvement due to the use of the grape family, which were consistent with the findings of the present study. 

In summary, previous studies demonstrated the improving effect of grape and its related products on memory and reported that the given effect was due to the antioxidant activity of phenolic compounds in grapes (Lamport et al., 2016 ▶; Lian et al., 2016 ▶; Farbood et al., 2016 ▶; Krikorian et al., 2012 ▶; Emami et al., 2010; Krikorian et al., 2010 ▶; Joseph et al., 2009 ▶). An animal study also reported the improving effect of “mavizˮ on memory which was suggested to be due to the antioxidant activity of non-phenolic compounds in “mavizˮ such as 5-hydroxymethylfurfural (5-HMF) which is negligible in fresh grapes while “mavizˮ is a good source of furfural (Bakhtiyari et al., 2017 ▶). 

It seems that the memory improvement induced by “mavizˮ consumption, is associated with the presence of both phenolic and non-phenolic compounds. 

In addition to research on the mechanism of the effect of the grape family on improving memory, ITM also provides explanations for the positive effect of “mavizˮ on memory. 

Based on ITM, all materials have a quality called “temperamentˮ by which the properties of that substance can be found. Each organ in the human body also has a unique temperament by which their specific needs for better performance and disease prevention, can be understood. According to ITM scholars such as Avicenna, the most common cause of amnesia and memory impairment is an increase in coldness and humidity of the brain temperament (Avicenn, 2008 ▶; Nazem Jahan, 2008 ▶). Therefore, memory improvement and treatment of forgetfulness require foods and medicinal herbs with warm temperament. This means that improvement in the performance of acoustic and visual memory requires the use of warm-tempered substances. As a result, “mavizˮ which has a warm and wet temperament in grade one (Hakim Moemen, 2011 ▶) and has been traditionally prescribed for memory improvement and treatment of dementia (Bakhtiyari et al., 2017 ▶) was used in this study. Accordingly, improvement of working memory could probably result from the effect of “mavizˮ on brain temperament. This change in brain temperament and consequently memory improvement caused by “mavizˮ consumption, could be possibly due to changes in the areas related to cognition and memory functions in the brain including middle temporal gyrus, cortex, and parts of the hippocampus reported in various studies following the use of grape and its related products (Gol et al., 2019 ▶; Bakhtiyari et al., 2017 ▶; Lamport et al., 2016 ▶; Krikorian et al., 2012 ▶; Krikorian et al., 2010 ▶).

Our findings revealed that the increase in visual memory span was non-significant. This might probably indicate that the brain needed a material warmer and drier than “mavizˮ, or the period of treatment and the amount of administered “mavizˮ should be increased significantly improve the visual memory span. Likewise, the non-significant increase of visual memory span might be due to the visual coding property of the brain, in which the visual codes could disappear faster than acoustic ones (Atkinson and Nolen-Hoeksema, 2014 ▶).

ITM has also prescribed herbal drugs for dementia with warm and dry temperament and higher grades than “mavizˮ (Aghili Alavi khorasani, 2009 ▶; Hakim Moemen, 2011 ▶; Nazem Jahan, 2008 ▶) while “mavizˮ is a medicinal food with both food and medicinal values (At -tabib Esfarayeny, 2014 ▶). 

Considering the result of the N-back Task, the non-significant increase in the percentage of true responses and decrease in the mean reaction time in the second and third levels, might be due to the ability of study subjects to learn the tests. Moreover, application of a substance warmer and drier than “mavizˮ or extending the period and increasing the amount of “mavizˮ consumption might significantly improve the percentage of true responses and the mean reaction time in the second and third levels.

The N-Back Task requires a higher level of attention and maintenance as well as manipulation and retrieval of information compared with the Digit Span Task. In this study, the results of the N-Back Task demonstrated that “mavizˮ was likely to cause the person to interpret and synchronize digits more accurately after receiving the data. It means that “mavizˮ could affect higher levels of attention, maintenance and manipulation, as well as information retrieval. Therefore, memory improvement at this level might be due to a change in the brain temperament.

In ITM, memory consists of several parts. one of which is related to analysis, interpretation, and manipulation of data (Avicenn, 2008 ▶). It seems that “mavizˮ can affect at this part.

Considering the complications observed in the study subjects, ITM has mentioned that “mavizˮ consumption may have side effects in individuals with high-grade warm and dry temperament (Hakim Moemen, 2011 ▶). For example, insomnia, headache, acne, and hematuria indicate excessive warming of the brain and other body parts.

In this preliminary study, there were biases and methodological restrictions that must be considered in future investigations. It should be noted that the ability to learn memory tests by individuals could lead to improved results in the control group. No placebo group was considered in this study because no material similar to “mavizˮ could be prepared and it was decided to use a natural type of “mavizˮ. However, the positive effect of “mavizˮ on memory improvement had been confirmed in rats (Bakhtiyari et al., 2017 ▶). Moreover, the diet of the study subjects could affect memory according to ITM while no specific restrictions were made on the diet of individuals in both groups. The authors could not also control stress in the subjects during the study. Accordingly, long-term memory was not assessed while it might have been changed by “mavizˮ consumption. 

The results of this randomized open-label clinical trial indicated that “mavizˮ might improve working memory. According to ITM data on the therapeutic and nutritional value of “mavizˮ, it was suggested to use “mavizˮ in the morning in healthy individuals. Furthermore, it was recommended to conduct an fMRI of working memory during the N-Back Task following the trial of “mavizˮ consumption. It is also recommended to conduct a clinical trial to assess the effect of “mavizˮ and other grape-related products on memory improvement and to compare the effects of these products. Due to the few side effects of “mavizˮ, it is suggested to conduct a clinical trial to assess the effect of “mavizˮ on memory improvement in patients suffering from AD.
